# Effect of Organic Manure on Crop Yield, Soil Properties, and Economic Benefit in Wheat-Maize-Sunflower Rotation System, Hetao Irrigation District

**DOI:** 10.3390/plants13162250

**Published:** 2024-08-13

**Authors:** Na Zhao, Jun Ma, Linmei Wu, Xiaohong Li, Hongwei Xu, Jun Zhang, Xiquan Wang, Yongqiang Wang, Lanfang Bai, Zhigang Wang

**Affiliations:** 1 College of Agronomy, Inner Mongolia Agricultural University, Hohhot 010019, China; 2 Bayannur Academy of Agricultural and Animal Sciences, Linhe 015400, China; 3 Inner Mongolia Hetao Irrigation District Water Conservancy Development Center, Linhe 015000, China; 4 Inner Mongolia Academy of Agricultural & Animal Husbandry Sciences, Hohhot 010031, China

**Keywords:** manure, soil properties, crop rotation, economic income, sustainable agriculture, crop yield

## Abstract

The combined application of manure and mineral fertilizer represents an effective strategy for enhancing crop yield. However, the relationship between soil fertility and crop yield remains unclear in saline-alkaline soil. Here, a 9-year field experiment (2015–2023) was conducted to investigate the effects of manure application and crop rotations on crop yield and economic efficiency as well as potential associated mechanisms in the Hetao Irrigation District. The results showed that in the third cropping rotation cycle, combined application of manure and mineral fertilizers (NPKO) caused a 6.2%, 38.9%, 65.3%, and 132.2% increase in wheat, sunflower, wheat equivalent yield, and the economic income of sunflower, respectively. The average grain yield had a positive correlation with soil organic matter and nutrient supply. This suggested that the soil organic matter had a positive effect on the crop yield due to its impact on nutrient supply. Simultaneously, the sunflower seed setting rate increased by 65.2% under NPKO. The linear regression model revealed that each additional input of 20 Mg ha^−1^ of manure resulted in an increase of 3.56 kg ha^−1^ in crop phosphorus harvest and a 0.05 Kg ha^−1^ increase in wheat equivalent yield compared to NPK. In conclusion, our results highlighted that manure application promotes soil properties and improves crop yield.

## 1. Introduction

The attainment of sustainable agricultural intensification is crucial in order to meet the growing demand for food. In order to adequately cater to the burgeoning population, projected to exceed 10 billion by the year 2100 [1], there needs to be a significant 48.6% surge in food production on a global scale. In modern agricultural practices, however, the excessive use of mineral fertilizers not only leads to widespread pollution of soil, atmosphere, and water resources, but also leads to a decline in soil properties and food production [2,3]. The recycling of agricultural organics, an age-old yet highly pertinent technology, has gained increasing popularity as a cost-effective means of nutrient management. This technology effectively maintains soil productivity while reducing reliance on inorganic fertilizers, aligning with the ongoing pursuit of sustainable agricultural practices. [4,5]. Numerous studies have confirmed that the combined application of manure and chemical fertilizers not only significantly improves crop yields in the short term [6,7], but also contributes to the enrichment of soil nutrients, such as soil organic matter and alkaline hydrolyzed nitrogen, in the long term [8]. For example, it was found that in semiarid areas, the joint application of manure and inorganic fertilizer increased yield quickly [9]. A study conducted in the south of China found that the application of sheep manure increased soil ammonium nitrogen content and alleviated soil acidification [10]. However, while manure has shown considerable potential in agricultural production, its application in specific environments, such as saline-alkaline soil, still faces a number of challenges. Saline-alkaline soil is a key constraint in food production, since the high salinity and alkalinity affect soil properties and nutrient cycling [11]. Currently, most research on the application of manure to saline-alkaline soils focus on single crops [11], and research involving long-term experiments and multi-rotation situations is even more lacking. Long-term experiments help us to gain a deeper understanding of the effect of manure on soil salinity and the trend of crop yield [12,13], while manure application in multi-rotation systems relates to how to effectively improve soil nutrients and enhance the nutrient uptake efficiency of crops. Therefore, more in-depth research on manure application in specific environments, such as saline-alkaline soil, is important for promoting the sustainable development of agricultural production.

The Hetao Irrigation District, situated in the city of Bayannur in Inner Mongolia, within the Yellow River basin, is the main production area for wheat, maize, and sunflower in China [14], and provides a solid support for guaranteeing Chinese national food security. Cultivated land in Bayannur is generally saline-alkaline soil, and the existing saline-alkaline cultivated land area is 3.2 × 10^4^ ha, accounting for about 36% of the total cultivated land area [15]. The vast tracts of saline and alkaline terrain within the city, which are widespread and exhibit poor soil properties, have emerged as a considerable impediment to crop production [16]. In addition, most farmers in Bayannur still persist in the habit of applying chemical fertilizers and organic fertilizers. In 2021, the cumulative chemical fertilizer input in Bayannur amounted to 4.27 × 10^10^ kg [17], and the soil was seriously compacted, with a large amount of loss of nitrogen fertilizer, which lead to a decline in soil properties and a rise in the pollution of clean water sources [16,18]. Sheep manure, as a by-product of the main farming industry in the Hetao Irrigation District, is abundant and easily accessible. However, the current problem of breeding separation not only wastes this valuable agricultural resource, but also may cause some pollution to the environment. Compared with the manure utilization rate of 50–70% in developed countries, the manure utilization rate of 30% in China leaves considerable potential for optimization and enhancement [2]. In 2020, the area of cultivated land with manure application in Bayannur was stabilized at more than 2.3 × 10^5^ ha [15]. Nonetheless, even with the extensive utilization of manure, research on its application specific to the saline and alkaline soil characteristics of the region remains inadequate. There is a noticeable absence of comprehensive scientific investigation and structured technical guidance in this area. To some extent, this limitation hinders the ability of manure to fully enhance soil properties and boost crop yields. It is imperative to intensify research efforts on the application of manure specifically tailored to this region. Therefore, a more comprehensive evaluation of the effects of fertilizer application on soil properties, crop yield, and economic benefits are required with respect to agronomic and environmental perspectives. A comprehensive analysis would facilitate us to understand the potential consequences of agricultural management strategies for the sustainable development of farmland.

Long-term experiments serve as pivotal indicators of sustainability and function as an early warning system to reveal problems that threaten future productivity. Therefore, the aim of this study was to provide a comprehensive account of the effects of the combined application of organic manure (sheep manure) and chemical fertilizers on spring wheat, maize, and sunflower yields, soil properties, and economic incomes in a three-crop rotation experiment. Herein, we conducted a long-term field experiment (from 2015 to 2023) to evaluate the effects of fertilizer strategy on crop yields, soil properties, and economic benefits in the diversified wheat-maize-sunflower rotation. We hypothesized that: (1) the combined application of manure and mineral fertilizers (NPKO) would result in a synergistic effect, leading to higher crop yields than those achieved through the individual application of mineral fertilizer (NPK); (2) the integration of manure into the fertilization strategy would enhance soil properties by improving soil nutrient availability, thereby contributing to the increased crop yields; and (3) the economic benefits of the combined fertilization strategy would counterbalance the costs, resulting in higher economic incomes for farmers. The findings of this study are expected to provide valuable insights into optimal fertilization strategies for sustainable crop production, taking into account both yield and economic considerations.

## 2. Results

### 2.1. Soil Properties

**Figure 1:**
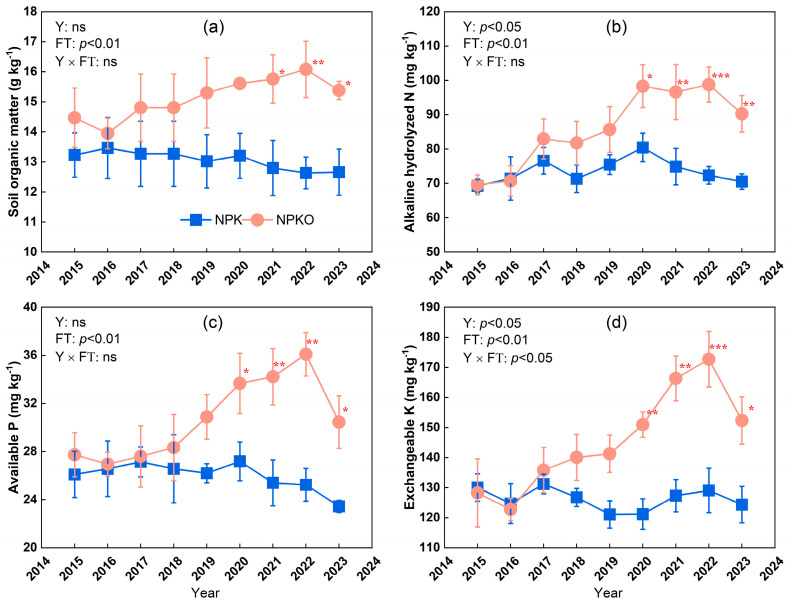
Values are means ± standard errors (*n* = 3). The content of soil organic matter (**a**), alkaline hydrolyzed N (**b**), available P (**c**), and exchangeable K (**d**) as affected by fertilization strategies from 2015 to 2023. The *p* values were obtained after a two-way ANOVA for Y (year) and FT (fertilizer treatments), where ns indicates no significant differences. * Indicates significant differences at the *p* < 0.05 level with and without organic manure; ** indicates significant differences at the *p* < 0.01 level; *** indicates significant differences at the *p* < 0.001 level.

In 2020, the input of manure improved the soil physicochemical properties (Figure 1; *p* < 0.05). The soil organic matter contents under fertilization with NPKO were 15.8 mg kg^−1^, 16.1 mg kg^−1^, and 15.4 mg kg^−1^ in 2021–2023, respectively, which were significantly higher than that under NPK (*p* < 0.05). Similarly, NPKO increased the soil alkaline hydrolyzed nitrogen, available phosphorus, and exchangeable potassium contents relative to NPK in 2020–2023. Specifically, the soil available nitrogen values under NPKO were 98.3 mg kg^−1^, 96.6 mg kg^−1^, 98.8 mg kg^−1^, and 90.2 mg kg^−1^, while the soil available nitrogen contents under NPK were 80.5 mg kg^−1^, 74.9 mg kg^−1^, 72.3 mg kg^−1^, and 70.5 mg kg^−1^, respectively, from 2020 to 2023. The available phosphorus contents under NPKO were 33.7 mg kg^−1^, 34.2 mg kg^−1^, 36.1 mg kg^−1^, and 30.5 mg kg^−1^, whereas the available phosphorus contents under NPK were 27.2 mg kg^−1^, 25.4 mg kg^−1^, 25.2 mg kg^−1^, and 23.4 mg kg^−1^, respectively.

### 2.2. Yield and Yield Components

**Figure 2:**
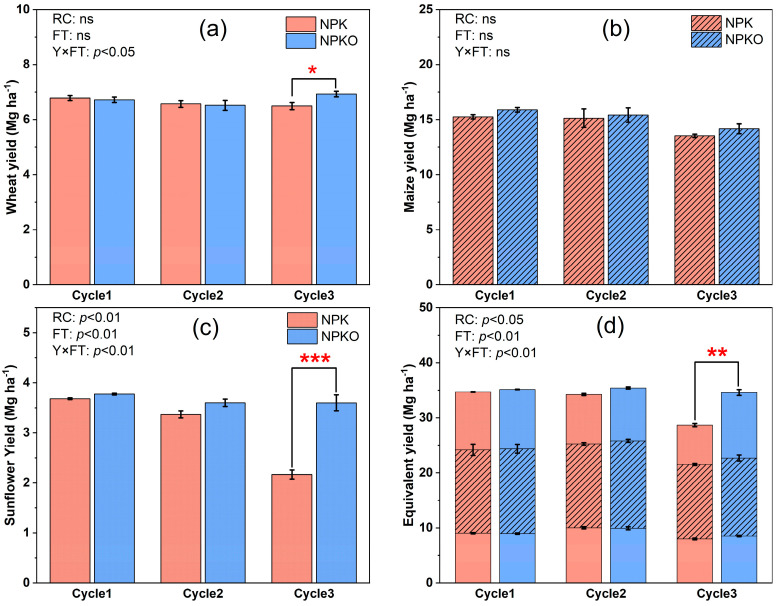
Values are means ± standard errors (*n* = 3). The grain yields of (**a**) spring wheat, (**b**) maize, and (**c**) sunflower, as well as (**d**) the maize equivalent yield in the wheat-maize-sunflower rotation system from 2015 to 2023, as affected by fertilization strategies. The *p* values were obtained after a two-way ANOVA for RC (rotation cycle) and FT (fertilizer treatment), where ns indicates no significant differences. * Indicates significant differences at the *p* < 0.05 level with and without organic fertilizer; ** indicates significant differences at the *p* < 0.01 level; *** indicates significant differences at the *p* < 0.001 level.

There was no difference in the crop yield in the first and second rotations, regardless of crop types (*p* > 0.05). In the third rotation cycle, wheat yield was significantly greater under NPKO (6.9 Mg ha^−1^) than under NPK (6.5 Mg ha^−1^; Figure 2). Similarly, sunflower yield was around 3.6 Mg ha^−1^ under NPKO, which was significantly higher than that under NPK (2.2 Mg ha^−1^). However, organic fertilization did not alter the yield of maize in the third rotation (*p* > 0.05). Moreover, wheat equivalent yield under NPKO was significantly larger than that under NPK in the third rotation cycle (11.9 Mg ha^−1^ vs. 7.2 Mg ha^−1^). In addition, crop rotation, fertilizer application, and their interaction significantly affected the wheat equivalent yield of crop.

Application of manure and crop rotation did not affect the yield components and above-ground biomass of wheat (*p* > 0.05), whilst crop rotation significantly changed the harvest index of wheat (Table 1). Although the 100-kernel weight of maize decreased with the crop rotation cycles (*p* < 0.05), crop rotation and fertilizer application did not affect the ear number, above-ground biomass of maize, or the harvest index (*p* > 0.05). Crop rotation significantly affected yield components, the above-ground biomass, and the harvest index of sunflower (*p* > 0.05). Specifically, NPKO increased seed number and seed setting rate by 20.1% and 65.2% as compared to NPK in the third crop rotation cycle, respectively (*p* < 0.05). Additionally, the interaction of fertilization and crop rotation significantly affected the seed setting rate of sunflower (Table 1).

### 2.3. Correlation between Soil Properties, Yield Components, and Crop Yield

**Figure 3:**
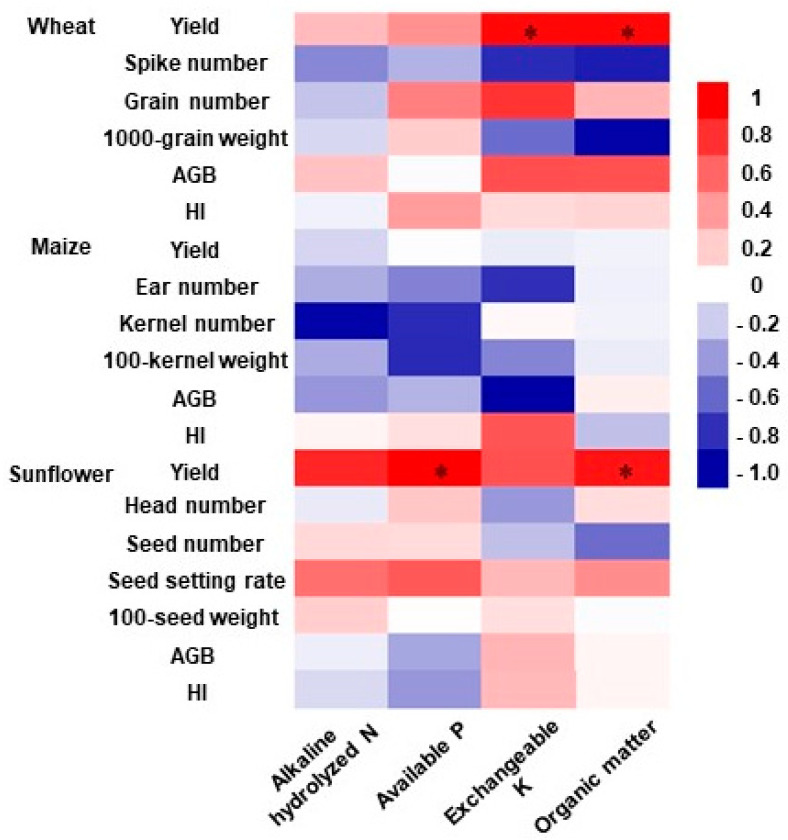
Correlation between yield components and soil nutrients. * Indicates significant differences at the *p* < 0.05 level. AGB: aboveground biomass; HI: harvest index; N: nitrogen; P: phosphorus; K: potassium.

Pearson’s correlation analysis showed that both the aboveground biomass and harvest index of wheat and maize were positively correlated with wheat and maize yield (*p* < 0.05; Table 2; Figure 3). The kernel number of maize was positively correlated with maize yield, whilst the seed setting rate of sunflower showed a positive correlation with sunflower yield. There was also a positive association between exchangeable potassium and soil organic matter with wheat yield (*p* < 0.05). Similarly, sunflower yield was positively related to effective phosphorus and soil organic matter (*p* < 0.05).

**Figure 4:**
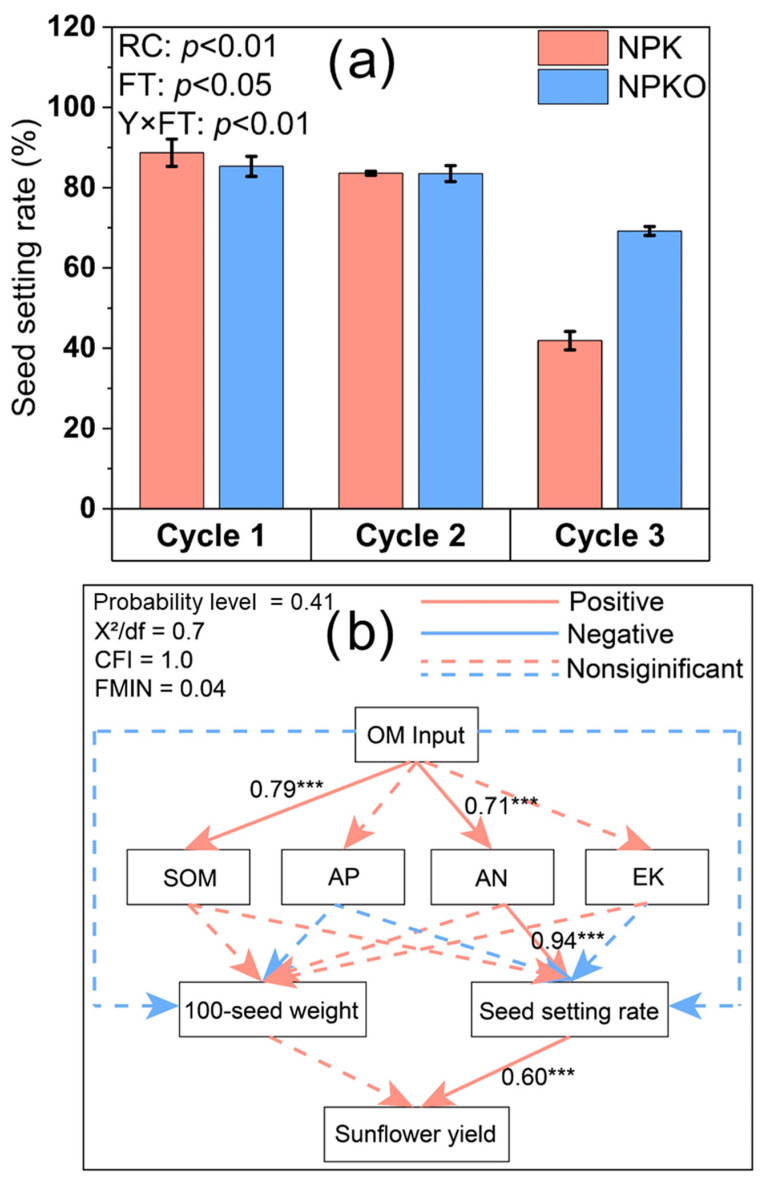
(**a**) Sunflower seed setting rate; (**b**) structural equation modelling of the effect of organic matter input on sunflower yield. OM: organic manure; AN: alkaline hydrolyzed nitrogen; AP: available phosphorus; EK: exchangeable potassium. *** indicates significant differences at the *p* < 0.001 level.

**Figure 5:**
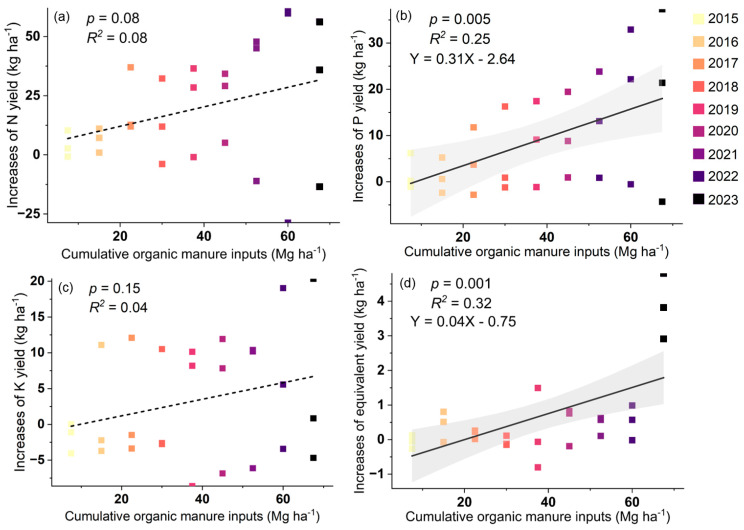
Linear regression of increases in N (**a**)/P (**b**)/K (**c**) yield and cumulative organic manure inputs (**d**). N: nitrogen; P: phosphorus; K: potassium. Grey areas are the 95% confidence intervals of linear regression models.

Structural equation modelling (SEM) explained 90.7% of the sunflower yield variation (Figure 4). Manure inputs increased sunflower seed setting rate by increasing soil alkaline hydrolyzed nitrogen content, and subsequently resulted in increased sunflower yield. The cumulative input of manure was positively correlated with the cumulative content of phosphorus harvested (Figure 5; R^2^ = 0.25, *p* = 0.005). Moreover, the cumulative input of manure was positively correlated with the cumulative content of the equivalent yield variation (R^2^ = 0.32, *p* = 0.001).

### 2.4. Economic Benefits

The cost of applying manure was greater than that of applying mineral fertilizer, with maize having the highest cost at ¥12,130 ha^−1^ (Table 3). In the first and second rotation cycles, the net income under manure was less than that under mineral fertilizer for all three of wheat, maize, and sunflower. However, in the third rotation, the net income for maize and sunflower were around ¥24,701 and ¥19,105 ha^−1^, respectively, under NPKO, which was ¥150 and ¥10,804 ha^−1^ greater compared with NPK (*p* < 0.05, Table 3).

## 3. Discussion

### 3.1. Soil Properties and Crop Yield

The positive effect of the combined mineral fertilizers with manure on soil properties agrees with field observations from long-term experiments given the positive impacts of manure on the structure and nutrient retention capacity of soils [19,20]. While our study did not directly investigate microbial activity or enzyme profiles, we acknowledge the importance of these factors in mediating soil property improvements under organic manure. A study performed in the Loess Plateau of China observed that an increase in soil properties was probably related to stimulated nutrient activation caused by the greater soil microbial and enzymatic activity under organic manure over time [21,22]. Additionally, sheep manure contains many functional groups that can bind to soil organic molecules and promote polymerization to form soil aggregates, which ultimately contributes to a stable soil organic matter content [23]. A study in northwest China found that manure also provides essential macro- and micronutrients and improves soil microbial activity [24], thereby providing slow-release nitrogen in third-rotation crop growth [20]. Consequently, with an increasing number of crop rotation cycles and the application of sheep manure, the accumulation and enhancement of soil organic matter, as well as soil nutrient content, became significant.

**Figure 6:**
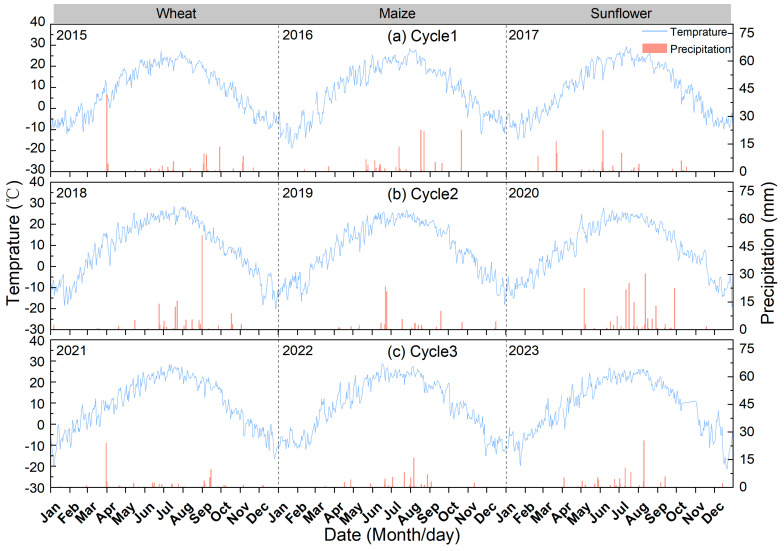
Daily precipitation (vertical bars) and mean air temperature (solid curves) in Cycle1 (**a**), Cycle2 (**b**) and Cycle3 (**c**). The spring wheat, maize, and sunflower seasons are labelled.

In the third rotation, NPKO significantly increased the yield of wheat and sunflower relative to NPK, which is consistent with our first hypothesis (Table 1, Figure 2). We admit that the higher yield under NPKO was expected given the additional nutrient supply. However, the response of crop yield to combined applications of manure and chemical fertilizer was absent in the first two crop rotations. The combined application of organic and mineral fertilizers significantly increased the crop yield in the third crop rotation [22]. The delayed yield response to manure was possibly due to the fact that the buffer time of achieving the minimum nutrient thresholds for limiting production was required in the depleted soil [25], which is consistent with changes in soil nutrient content over the crop rotation cycle. Notably, the application of manure was found to be more influential than crop rotation in enhancing crop yield, aligning with previous studies that reported positive effects on nitrogen use efficiency [26,27]. Additionally, the seed setting rate of sunflower significantly increased, whereas fertilizer application did not affect the yield components of maize and wheat. This suggests that the effect of manure varies among crops, probably due to the different growth characteristics and nutrient requirements of crops [27,28]. Moreover, the application of manure did not improve maize yield, and the rotation reduced the 100-kernel weight of maize. This could be attributed to lower precipitation during the maize cropping cycle (Figure 6; 116.9 mm → 89.6 mm → 68.8 mm), since soil water constitutes the primary limiting factor impeding maize growth in arid regions [29,30]. In other words, the application of manure may not alleviate the reduction in grain yields caused by water deficit.

### 3.2. Mechanisms That Affecting Yield under Organic Fertilization

Pearson’s correlation analysis revealed a strong link between crop yield and its associated growth parameters and soil nutrients (Figure 3 and Table 2). Exchangeable potassium and available phosphorus are essential nutrient elements for crop growth, and also play a key role in crop metabolism and physiological processes [31]. For example, it has been shown that sunflowers require large amounts of phosphorus before seed filling, and that the reuptake of phosphorus by the leaves and stems of mature sunflower achenes ranges from 30 to 60% [32]. Furthermore, studies have revealed that plants demonstrate enhanced efficiency in utilizing phosphorus when it is in a less abundant state, thereby maximizing their potential in utilizing the absorbed nutrient [19,33]. Therefore, chemical fertilizers are not applied as efficiently as low phosphorus utilizing fertilizers such as sheep manure, which results in higher phosphorus use efficiency, and an even higher yield for the crop.

Our SEM results showed that manure inputs increased sunflower yield by increasing soil alkaline hydrolyzed nitrogen, which in turn increased sunflower seed setting rate (Figure 4). This confirmed the second hypothesis. In general, the nitrogen contained in manure is not immediately accessible for absorption by plants and must be mineralized to convert it into inorganic nitrogen forms that are available for plant utilization [34,35]. The increase in soil alkaline hydrolyzed nitrogen could be therefore explained by the following reasons: (1) the addition of fertilizers may increase the net mineralization of soil organic nitrogen [36,37]; and (2) some studies have shown that improvements in microbial community structure under organic fertilizer [21,38] increase soil organic carbon turnover and organic mineralization, resulting in improvements in nutrient supply [39,40]. Nitrogen is an essential component of proteins and chlorophyll and is physiologically important in plant metabolism. An abundant supply of nitrogen is essential to promote all metabolic processes that are responsible for rapid crop growth and high yield. Thus, an increase in soil alkaline hydrolyzed nitrogen provides abundant nutrients to crops, thereby increasing the fruiting rate and leading to an increased yield of sunflower.

Furthermore, manure sustained soil phosphorus yield and wheat equivalent yield, and crop phosphorus yield increased by 3.56 kg ha^−1^ for each additional 20 Mg ha^−1^ of manure input, as evidenced by the linear regression analysis (Figure 5). This may be due to the fact that the combined application of organic and mineral fertilizers increased crop P utilization efficiency, which subsequently increased soil phosphorus content and phosphorus uptake, and consequently, phosphorus absorption in cereals. In addition, our study found that for each 20 Mg ha^−1^ increase in manure input, the wheat equivalent yield increased by 0.05 kg ha^−1^. These findings shed light on the specific mechanism by which organic manure influences crop yield, providing a theoretical foundation for optimizing fertilizer application programs.

### 3.3. Economic Income

Economic income is an important factor that cannot be ignored in agricultural production [41]. Despite the high initial input cost of manure application, its economic benefits gradually increased in the long-term crop rotation (Table 3). For example, in the third crop rotation cycle, NPKO had significantly higher net income both in maize and sunflower season than NPK. In crop cultivation, the utilization of manure affects yield, quality, the cost of agriproducts, and the economic subsidy acquisition of farmers. Economic benefits would motivate farmers to adjust planting decisions. Although the input of manure increased the initial cost, it improved crop yield and quality in the long-term, which in turn increased farmers’ income, as well as achieving sustainable development of agriculture [41]. It should be noted that the transition to green production will inevitably bring some pressure to farmers by way of reducing the economic income of farmers in a certain range [42]. On the contrary, the greater the farmers’ social responsibility for the environment, the more obvious positive feedback to cropping behavior will be (e.g., mitigating soil degradation, reducing carbon sinks and N_2_O emissions), which is beneficial for the sustainable development of agriculture, as well as bringing in more profitability. Therefore, it is imperative to prioritize the education and training of farmers, enhancing their technical proficiency and management capabilities in fertilizer application, to guarantee that the full potential of manure application is realized [43]. Taking the build-up and maintenance of soil organic matter under organic fertilizer, as well as its supporting effects on fertilizer use efficiency, into consideration, it may be easier for farmers to adjust their agronomic management [44], and they may prefer to combine the use of these inputs. In short, our study seems to highlight that a minimum of 9 years was required for organic fertilizers in the wheat-maize-sunflower rotation system in the Hetao Irrigation District to surpass the yield generated under mineral fertilizer application.

## 4. Materials and Methods

### 4.1. Site Description

This long-term field trial was located at the experimental base of Yuanzi Drainage of Bayannur Academy of Agricultural and Animal Husbandry Sciences (40°90′ N, 107°17′ E). The experimental base is located in the hinterland of the Hetao Irrigation District, with an altitude of 1035 m. The average annual temperature is 3.7–7.6 °C, the annual sunshine hours are 3100–3300 h, the effective cumulative temperature is 2900–3200 °C, and the frost-free period is 130–150 d. The annual precipitation ranges from 139 to 222 mm, and the annual evaporation is 2030–3180 mm (Figure 6), which is typical of a mid-temperate continental monsoon climate. The soil was silt loam with a soil organic matter content of 13.0 g kg^−1^ (0–20 cm), the available nitrogen (N) concentration was 73 mg kg^−1^, the available phosphorus (P) concentration was 26.2 mg kg^−1^, the exchangeable potassium (K) concentration was 130.0 mg kg^−1^, the soil total water-soluble salt value was 0.58 g kg^−1^, and the soil pH was 8.8 (Table 4).

### 4.2. Experimental Design

The experiment was conducted in a spring wheat-spring maize-sunflower crop rotation system from 2015 to 2023. Specifically, spring wheat was planted in 2015, 2018, and 2021; maize in 2016, 2019, and 2022; and sunflowers in 2017, 2020, 2023. The experiment contained two treatments: NPK (mineral fertilizer) and NPKO (mineral fertilizer + sheep manure), with three replications, arranged in randomized blocks. The plot area was 40 m^2^, with a length of 10 m and width of 4 m. The fertilizer NPK application rate for both treatments was: N: 225 kg ha^−1^; P_2_O_5_: 120 kg ha^−1^; and K_2_O: 90 kg ha^−1^. All of the phosphorus fertilizer was used as base fertilizer; 70% of the potassium fertilizer was used as base fertilizers, and 30% was used as top dressing combined with irrigation (wheat and maize in the pulling out stage, sunflower in the bud stage); and 30% of the nitrogen fertilizer was used as base fertilizer, while 70% was used as top dressing combined with irrigation (wheat and maize in the pulling in stage, sunflower in the bud stage). Nitrogen, phosphate, and potassium fertilizers were applied as urea, calcium superphosphate, and potassium chloride, respectively. Sheep manure was applied in the first year of the experiment during the wheat sowing before spreading rotary, and in the rest of the years it was applied in the autumn every year, before turning over the land to spread ploughing. The manure was folded dry at a volume of 7500 kg ha^−1^. The N, P_2_O_5_, and K_2_O contents of the sheep manure were 0.78%, 0.35%, and 0.89%, respectively. All straw was removed after harvesting. Both maize and sunflower were grown with plastic film mulching. Plough tillage was performed before irrigation the previous autumn, and the land was tilled to a depth of 20 cm with a rotavator before sowing. The experimental plots were all irrigated with Yellow River water using border irrigation, with 1875 m^3^ ha^−1^ for wheat, 2250 m^3^ ha^−1^ for maize, and 1125 m^3^ ha^−1^ for sunflower. Other field management conditions were similar, in alignment with local field practices.

The wheat variety was Yongliang 4, the maize variety was Simon 568, and the sunflower variety was edible sunflower hybrid SH363. Wheat was sown on 20 March, with a sowing rate of 375 kg ha^−1^, and harvested on 20 July; maize was sown on 25 April, with large and small rows planted in a mulch planting, with large rows of 65 cm, small rows of 35 cm, and plant spacing of 26.7 cm, and a density of 75,000 plants ha^−1^, and harvested before 1 October; sunflower was sown in late May, with large and small rows planted in mulch, with large rows of 80 cm and small rows of 35 cm, as well as plant spacing of 50 cm, density of 33,000 plants ha^−1^, and harvested in late September.

### 4.3. Sample Collection and Measurement

All the soil samples were collected with a 5 cm diameter corer (inner diameter: 4 cm) after crop harvest, and an ‘S’ shaped sampling method was used in each experimental plot. Three cores per plot were mixed together to minimize spatial heterogeneity. The soil samples were taken back to the laboratory as soon as possible. Air-dried soil was milled and passed through a 2 mm sieve for the measurement of pH, alkaline hydrolyzed nitrogen, available phosphorus (AP), exchangeable potassium (EK), and soil organic matter (SOM) contents. Soil pH was measured using an aqueous extraction method with a water:soil ratio of 2.5:1. Soil organic matter was determined by external heating with potassium dichromate; soil total nitrogen by the Kjeldahl method [45]; alkaline hydrolyzed nitrogen using reduction diffusing method [46]; soil available phosphorus by the sodium bicarbonate leaching-molybdenum antimony colorimetric method [47]; total P using the molybdenum–antimony colorimetric method; soil exchangeable potassium by ammonium acetate leaching-flame photometer method [48]; and total K by flame photometry.

The number of plants, seed yield, above-ground biomass, and harvest index of each plot were determined at maturity of wheat, maize, and sunflower. Plant samples were chopped and air-dried until a constant weight was reached. All plant samples were then separated into seed and straw and above ground biomass. Harvest index was determined as the ratio of seed biomass to above ground biomass at maturity. For each plot, 20 wheat, 5 maize, and 5 sunflower were selected to determine yield component, including grain number and grain weight.

Since the differences in the economic benefits of wheat, maize, and sunflower under fertilizer applications were dependent on crop yield, selling price, and labor cost, the net incomes were calculated as the difference between average inputs and outputs. Inputs included the costs of fertilizers, irrigation, herbicides, mixtures, seeds, and labor, and outputs contained the benefits of production.

### 4.4. Data Analysis

All data were expressed as means ± standard error (SE). The data that did not normally distribute were given log-transformation. One-way ANOVA was applied to evaluate the effect of fertilization on all of the tested parameters. Tukey’s post hoc test was used for multiple comparisons with a significance level of *p* < 0.05. We used structural equation modeling (SEM) to evaluate the direct and indirect effects of organic manure and soil properties on the concentration of the different P pools. The model has a good fit when χ^2^/df is ≤2, the probability level is >0.05, and RMSEA is indistinguishable from zero. All the figures were plotted in Origin 2021, and all the statistical analyses were conducted in SPSS 26.0 (IBM SPSS Software Inc., Armonk, NY, USA).

To assess the performance of the crop rotation systems, the grain yields of maize and sunflower were standardized to wheat equivalent yield [49]:

(1) $$\mathrm{Wheat}\;\mathrm{equivalent}\;\mathrm{yield}=\frac{Pnon-wheat}{Pwheat}\times \mathrm{Ynon}-\mathrm{wheat},$$

where Pnon-wheat is the price of non-wheat crops, Pwheat is the price of wheat, and Ynon-wheat is the yield of non-wheat crops. The average prices of wheat, maize, and sunflower were $0.45, $0.34, and $0.98 kg^−1^, respectively.

## 5. Conclusions

Based on a nine-year field experiment in the Hetao Irrigation District, our results showed that the combination of manure and mineral fertilizer significantly increased wheat, sunflower, and wheat equivalent yields by 6.2%, 38.9%, and 65.3%, respectively, in the third cropping rotation cycle (2020–2023). Additionally, the combined application of manure and mineral fertilizer increased soil organic matter and available nutrients, and as a consequence improved soil property. The enhancement in alkaline hydrolyzed nitrogen contributed to the improved sunflower seed setting rate and subsequent crop yield under organic fertilizers. Economic analysis revealed that sunflower grown under the combination of manure and mineral fertilizers caused 132.2% higher economic incomes compared to mineral fertilizer alone. Linear regression analysis further indicated that each additional 20 Mg ha^−1^ of manure resulted in an increase of 3.56 kg ha^−1^ in crop phosphorus harvest and 0.05 Kg ha^−1^ increase in wheat equivalent yield compared to NPK. Overall, the combined application of manure and mineral fertilizers was an effective approach to enhance crop yield and economic income while addressing soil degradation issues caused by the excessive use of mineral fertilizers. Therefore, we suggest that 20 Mg of manure per hectare of land could be added to the cultivated species in the Hetao Irrigation Distinct in northwest China to increase total equivalent crop yield.

## Figures and Tables

**Table 1:** The yield components, above ground biomass (AGB), and harvest index (HI) of spring wheat, maize, and sunflower as affected by fertilization strategies across the three cycles of the wheat-maize-sunflower rotation system from 2015 to 2023.

Treatments	Cycle 1 (2015–2017)		Cycle 2 (2018–2020)		Cycle 3 (2021–2023)		Two-Way ANOVA		
	NPK	NPKO	NPK	NPKO	NPK	NPKO	RC	FT	RC × FT
Wheat season (2015, 2018, 2021)									
Spike number (m^−2^)	720 ± 3	717 ± 3	728 ± 4	707 ± 18	724 ± 19	714 ± 5	ns	ns	ns
Grain number (spike^−1^)	34.7 ± 1.2	32.8 ± 2.1	36.4 ± 1.9	32.3 ± 1.4	35.4 ± 1.6	37.4 ± 2.5	ns	ns	ns
1000-grain weight (g)	53.9 ± 1.8 a	49.0 ± 3.7 b	50.1 ± 3.1	43.2 ± 0.7	49.6 ± 2.2	50.6 ± 0.8	ns	ns	ns
AGB (Mg ha^−1^)	13.3 ± 0.3	13.1 ± 0.3	13.6 ± 0.4	13.9 ± 0.2	12.7 ± 0.4	13.5 ± 0.2	ns	ns	ns
HI	0.51 ± 0.00	0.51 ± 0.00	0.48 ± 0.01	0.47 ± 0.01	0.51 ± 0.00	0.51 ± 0.00	**	ns	ns
Maize season (2016, 2019, 2022)									
Ear number (m^−2^)	7.40 ± 0.26	7.37 ± 0.12	7.20 ± 0.30	7.65 ± 0.04	7.12 ± 0.07	7.95 ± 0.20	ns	ns	ns
Kernel number (ear^−1^)	654 ± 29	686 ± 30	634 ± 19	660 ± 16	623 ± 29	608 ± 41	ns	ns	ns
100-kernel weight (g)	35.1 ± 0.7	35.6 ± 0.7	33.4 ± 0.2	35.7 ± 0.9	32.8 ± 0.5	33.2 ± 0.6	*	ns	ns
AGB (Mg ha^−1^)	33.2 ± 1.0	34.5 ± 0.5	31.8 ± 0.9	32.4 ± 0.5	29.9 ± 1.0	31.3 ± 1.0	ns	ns	ns
HI	0.46 ± 0.00	0.46 ± 0.00	0.47 ± 0.02	0.48 ± 0.03	0.46 ± 0.02	0.47 ± 0.02	ns	ns	ns
Sunflower season (2017, 2020, 2023)									
Head number (m^−2^)	3.01 ± 0.02	3.12 ± 0.07	3.00 ± 0.12	3.03 ± 0.03	2.63 ± 0.03	2.53 ± 0.03	**	ns	ns
Seed number (head^−1^)	970 ± 93	913 ± 20	1320 ± 32	1325 ± 43	1190 ± 117 a	990 ± 57 b	*	ns	ns
Seed setting rate (%)	88.7 ± 3.4	85.3 ± 2.5	83.6 ± 0.5	83.5 ± 2.0	41.9 ± 2.3 b	69.2 ± 1.1 a	**	*	**
100-seed weight (g)	17.7 ± 0.9	17.7 ± 0.2	23.4 ± 1.0	23.7 ± 0.8	27.4 ± 0.6	26.9 ± 0.2	**	ns	ns
AGB (Mg ha^−1^)	12.7 ± 0.2	13.1 ± 0.2	12.5 ± 0.5	13.8 ± 0.6	20.1 ± 0.6	20.8 ± 0.7	**	ns	ns
HI	0.29 ± 0.00	0.29 ± 0.01	0.27 ± 0.01	0.26 ± 0.01	0.30 ± 0.03	0.29 ± 0.00	ns	ns	ns

Different lowercase letters within a row of the same rotation cycle represent significant differences at *p* < 0.05 by the Fisher’s Least Significant Difference (LSD) test. Values are means ± standard errors (*n* = 3). RC, rotation cycle; FT, fertilizer treatment; and RC × FT, interaction between rotation cycles and fertilizer treatments. AGB: above ground biomass; HI: harvest index; *, *p* < 0.05; **, *p* < 0.01; and ns, non-significant difference. The below is the same.

**Table 2:** Pearson’s correlation coefficients between grain yield and yield components for spring wheat, maize, and sunflower.

Wheat Yield (2015, 2018, 2021)		Maize Yield (2016, 2019, 2022)		Sunflower Yield (2017, 2020, 2023)	
Spike number	−0.42	Ear number	0.43	Head number	0.47
Grain number	0.42	Kernel number	0.50 *	Seed number	−0.28
1000-grain weight	0.13	100-kernel weight	0.46	100-seed weight	−0.59 *
AGB	0.51 *	AGB	0.61 **	AGB	−0.55 *
HI	0.47 *	HI	0.55 *	HI	0.29
				Seed setting rate	0.89 **

Spike number, grain number, 1000-grain weight, aboveground biomass (AGB), and harvest index (HI) were tested for spring wheat. Ear number, kernel number, 100-kernel weight, AGB, and HI were analyzed for maize. Head number, seed number, 100-seed weight, AGB, HI, and seed setting rate were used for sunflower. *, *p* < 0.05; **, *p* < 0.01

**Table 3:** Inputs and outputs of wheat, maize, and sunflower as affected by fertilization strategies across three cycles of a wheat-maize-sunflower rotation system from 2015 to 2023.

Item	Spring Wheat		Maize		Sunflower		Price
	NPK	NPKO	NPK	NPKO	NPK	NPKO	
Average inputs							
Seed (kg ha^−1^)	375	375	45	45	30	30	¥5, 20, and 40 kg^−1^ for wheat, maize, and sunflower, respectively
Organic fertilizer (kg ha^−1^)	0	7500	0	7500	0	7500	¥0.1 kg^−1^
Urea (kg ha^−1^)	387	387	485	485	485	485	¥4 kg^−1^
Diammonium phosphate (kg ha^−1^)	261	261	261	261	261	261	¥3.7 kg^−1^
Potassium sulfate (kg ha^−1^)	180	180	180	180	180	180	¥3.3 kg^−1^
Herbicide (bottle ha^−1^)	15	15	22.5	22.5	22.5	22.5	¥20 bottle^−1^
Irrigation (m^3^ ha^−1^)	1875	1875	2250	2250	1125	1125	¥0.8 m^−3^
Plastic film mulching (kg ha^−1^)	0	0	50	50	37.5	37.5	¥12 kg^−1^
Labor (No. ha^−1^)	0	0	0	0	5	5	¥150 labor^−1^
Machinery (times year^−1^)							
Rotary tillage	1	1	1	1	1	1	¥675 ha^−1^
Soil rolling	1	2	1	2	1	2	¥750 ha^−1^
Sowing	1	1	1	1	1	1	¥750 ha^−1^
Harvest	1	1	1	1	1	1	¥750, 1200, and 900 ha^−1^ for wheat, maize, and sunflower, respectively
Plough tillage	1	1	1	1	1	1	¥750 ha^−1^
Total (¥ ha^−2^)	9713	11,213	10,630	12,130	10,330	11,830	
Average outputs, crop yield (Mg ha^−1^, 13%, 14% of wheat, maize moisture content)							
Cycle1 (2015–2017)	6.8	6.8	15.2	15.5	3.7	3.8	¥3200, 2100, and 6000 Mg^−1^ for wheat, maize, and sunflower, respectively
Cycle 2 (2018–2020)	6.6	6.7	15.1	15.3	3.7	4.1	¥3200, 2400, and 6400 Mg^−1^ for wheat, maize, and sunflower, respectively
Cycle 3 (2021–2023)	6.5	6.9	13.5	14.2	2.2	3.6	¥3200, 2600, and 8600 Mg^−1^ for wheat, maize, and sunflower, respectively
Net income (¥ ha^−1^)							
Cycle1 (2015–2017)	11,994	10,294	21,395	21,227	11,759	10,803	
Cycle 2 (2018–2020)	11,308	9643	25,683	24,859	11,220	11,210	
Cycle 3 (2021–2023)	11,061	10,958	24,551	24,701	8301	19,105	

**Table 4:** Main characteristics of the soil before the experiment.

Soil Depth (cm)	SOM (g kg^−1^)	TN (g kg^−1^)	TP (g kg^−1^)	TK (g kg^−1^)	AN (mg kg^−1^)	AP (mg kg^−1^)	EK (mg kg^−1^)	TWS Salt (g kg^−1^)	pH
0–20	13.0 ± 1.1	0.80 ± 0.3	0.40 ± 0.1	20.0 ± 2.1	73 ± 3.8	26.2 ± 2.2	130 ± 6.9	0.58 ± 0.1	8.8 ± 1.3
20–40	12.3 ± 0.8	0.73 ± 0.2	0.56 ± 0.1	17.5 ± 1.9	70 ± 4.2	21.5 ± 1.7	130 ± 7.1	0.52 ± 0.1	8.9 ± 0.9

Values are means ± standard errors (*n* = 3). SOM: soil organic matter; AN: alkaline hydrolyzed nitrogen; AP: available phosphorus; EK: exchangeable potassium; TN: total nitrogen; TP: total phosphorus; TK: total potassium; TWS: total water-soluble salt.

## Data Availability

All data generated or used during the study appear in the submitted article.
